# The Challenge of Long-Term Cultivation of Human Precision-Cut Lung Slices

**DOI:** 10.1016/j.ajpath.2021.10.020

**Published:** 2022-02

**Authors:** Eike B. Preuß, Stephanie Schubert, Christopher Werlein, Helge Stark, Peter Braubach, Anne Höfer, Edith K.J. Plucinski, Harshit R. Shah, Robert Geffers, Katherina Sewald, Armin Braun, Danny D. Jonigk, Mark P. Kühnel

**Affiliations:** ∗ Institute of Pathology, Lung Research Group, Hannover Medical School, Hannover, Germany; † Genome Analytics, Helmholtz Centre for Infection Research, Braunschweig, Germany; ‡ Fraunhofer Institute for Toxicology and Experimental Medicine, Hannover, Germany

## Abstract

Human precision-cut lung slices (PCLS) have proven to be an invaluable tool for numerous toxicologic, pharmacologic, and immunologic studies. Although a cultivation period of <1 week is sufficient for most studies, modeling of complex disease mechanisms and investigating effects of long-term exposure to certain substances require cultivation periods that are much longer. So far, data regarding tissue integrity of long-term cultivated PCLS are incomplete. More than 1500 human PCLS from 16 different donors were cultivated under standardized, serum-free conditions for up to 28 days and the viability, tissue integrity, and the transcriptome was assessed in great detail. Even though viability of PCLS was well preserved during long-term cultivation, a continuous loss of cells was observed. Although the bronchial epithelium was well preserved throughout cultivation, the alveolar integrity was preserved for about 2 weeks, and the vasculatory system experienced significant loss of integrity within the first week. Furthermore, ciliary beat in the small airways gradually decreased after 1 week. Interestingly, keratinizing squamous metaplasia of the alveolar epithelium with significantly increasing manifestation were found over time. Transcriptome analysis revealed a significantly increased immune response and significantly decreased metabolic activity within the first 24 hours after PCLS generation. Overall, this study provides a comprehensive overview of histomorphologic and pathologic changes during long-term cultivation of PCLS.

The concept of using tissue slices to study organ metabolism was introduced by Otto Warburg as early as the 1920s.^1^^,^^2^ Since then, methods have improved markedly. The introduction of agarose filling^3^ and the Krumdieck microtome^4^ allowed for a more standardized generation and cultivation of so-called precision-cut lung slices (PCLS). PCLS are widely used as an *ex vivo* organ slice culture system for numerous toxicologic, immunologic, and pharmacologic studies in the respiratory system.^5^, ^6^, ^7^, ^8^, ^9^, ^10^, ^11^, ^12^, ^13^, ^14^, ^15^, ^16^, ^17^ They are an invaluable model to study the mechanisms of pulmonary fibrosis^18^, ^19^, ^20^ and asthma^21^^,^^22^ and to evaluate novel asthmatic^21^^,^^23^, ^24^, ^25^ and fibrosis therapies,^26^^,^^27^ even though the model phenotype may display characteristics different from the clinical ones.^28^ While most studies have been performed on rodent PCLS, for the sake of translatability, human PCLS are the better choice because modes of action and metabolism differ among species.^29^^,^^30^ The benefit of PCLS as a model is that a single lung can provide hundreds of slices for both control and experimental conditions while mimicking most of the structural and functional aspects of the respiratory tract and including all the relevant cell types, except those recruited by the circulatory system.^5^^,^^12^^,^^14^^,^^15^^,^^31^, ^32^, ^33^

Typically, PCLS are cultured for <1 week, which is suitable for the investigation of most toxicologic and pharmacologic outcomes^7^^,^^17^^,^^34^^,^^35^ but not for long-term investigation of repeated exposure to a substance or disease modeling. Indeed, recent studies found that human PCLS are viable, preserve structural integrity, contain all lung-relevant cell types, and are metabolically active for up to 15 days of cultivation.^12^^,^^13^^,^^36^ Additionally, the embedding of PCLS into specifically engineered hydrogels is able to significantly increase viability and pneumocyte type 2 functionality throughout 21 days of cultivation when compared with nonencapsulated controls.^37^ Human PCLS show cytotoxic, inflammatory, and immune responses to selected stimuli (eg, lipopolysaccharide and vaccine antigens) when cultured for up to 14 days.^12^ Even though the data are promising and point to a long-term experimental window for human PCLS cultivation,^12^ some functional and cellular changes are observed during long-term cultivation.^5^^,^^12^^,^^13^^,^^36^ Long-term cultivated PCLS show decreased cytokine and chemokine production in response to external stimuli, such as lipopolysaccharides.^12^^,^^13^ In addition, proteomic data indicate a persistent inflammatory activity during long-term cultivation, especially during the first 4 days in culture.^36^ Histomorphologically, a reduced number of pneumocytes and a slight degradation of connective tissue fibers have been described.^13^ Even though important aspects of long-term cultivation of PCLS was elucidated, quantitative, histologic analysis of tissue integrity is lacking.

In this study, human PCLS with diverse pathologic background and varying processing times were cultivated for up to 28 days to extensively quantify ongoing cellular and structural changes with an internally developed ranking system, evaluating the integrity of the alveolar and bronchiolar epithelium as well as the endothelium. Furthermore, viability, cilia functionality, and RNA sequencing (RNASeq) analysis conducted for the identification of altered pathways were analyzed. The aim of this study was to complement existing data of PCLS long-term cultivation by providing a detailed insight regarding the preservation of different compartments in tissue slices and analyzing histopathologic changes that occur during cultivation.

## Materials and Methods

### Human Samples

Human PCLS were generated from seven fibrotic lung explants (four women and three men; means ± SD age, 53 ± 10 years) during lung allograft transplantation and from nine lung tumor resections (three women and six men; means ± SD age, 67 ± 7 years) derived from patients with primary pulmonary adenocarcinoma (*n* = 5) and squamous cell carcinoma with cornification (*n* = 2) and from metastases from patients with invasive bladder carcinoma (*n* = 1) and thyroid carcinoma (*n* = 1) (Table 1). The cohort of fibrotic lung explants included two lungs with manifest idiopathic pulmonary fibrosis with a usual interstitial pneumonia pattern, one fibrotic lung with a nonspecific interstitial pneumonia pattern, three fibrotic lungs with usual interstitial pneumonia pattern in which the underlying disease was not conclusively determined, and one lung with end-stage fibrotic sarcoidosis. Lung tumor resection tissue that was used for PCLS generation was confirmed as tumor-free by an experienced pathologist (C.W. under the supervision of D.D.J.). All tissue donors provided informed consent for participating in the study, which was approved by the Hannover Medical School Ethics Committee (ethic vote 8867_BO_K_2020).

**Table 1:** Background Information of the Human Donors Used for the Generation of Precision-Cut Lung Slices

Donor no.	Sex	Age, years	Lung lobe	Weight, length × width × height	Diagnostic finding	Smoker status	Methods and analysis	Processing	Chemotherapy or radiotherapy
1	Male	71	LL, right	29 g, 13 × 4.5 × 2 cm	Filiae of a non–small cell carcinoma with low differentiation after bladder cancer	No smoking	WST-1 and LDH assay, cilia check, histologic evaluation, RNA sequencing	Immediate	No
2	Male	55	UL, left	7 × 3 × 3.5 cm	NSCLC after anaplastic thyroid cancer	60 py until 6 years before surgery	WST-1 and LDH assay, cilia check, histologic evaluation	Immediate	Radioiodo-therapy
3	Female	71	UL, left	155 g, 15 × 10 × 3 cm	Squamous cell carcinoma with low cornification and low differentiation	20 py until 6 years before surgery	WST-1 and LDH assays, cilia check, histologic evaluation	Immediately filled, then stored overnight at 4°C, before further processed	No
4	Female	75	LL, left	30 g, 10 × 6 × 3 cm	Adenocarcinoma	Until 16 years before surgery	WST-1 and LDH assays, cilia check, histologic evaluation	Immediate	No
5	Female	76	UL, left	18 × 12 × 6 cm	NSCLC, adenocarcinoma	No smoking	WST-1 and LDH assays, cilia check, histologic evaluation	Immediately filled, then stored overnight at 4°C, before further processed	No
6	Male	69	UL, right	131 g, 14 × 8.5 × 3 cm	Adenocarcinoma	75 py	WST-1 and LDH assays, cilia check, histologic evaluation	Immediate	No
7	Male	68	UL, left	589 g, 24 × 15 × 7 cm	NSCLC, adenocarcinoma with low differentiation	No smoking	WST-1 and LDH assays, cilia check, histologic evaluation, RNA sequencing	Immediate	No
8	Male	57	LL, left	493 g, 18 × 13.5 × 4.6 cm	Invasive adenocarcinoma	20 py	WST-1 and LDH assays, cilia check, histologic evaluation, RNA sequencing	Immediate	No
9	Male	58	UL, right	300 g, 19 × 15 × 3.5 cm	Squamous cell carcinoma with cornification	110 py	WST-1 and LDH assays, cilia check, histologic evaluation	Immediate	No
10	Female	62	LL, ML, right	565 g, 22 × 12 × 4.5 cm	Fibrosis, sarcoidosis	15 py until 19 years before surgery	Histologic evaluation	Immediately filled, then stored overnight at 4°C, before further processed	
11	Female	55	UL, LL, right	498 g, 19.5 × 12 × 6 cm	Fibrosis, UIP pattern, IPF	No smoking	WST-1 and LDH assays, cilia check, histologic evaluation	Tissue stored overnight at 4°C, then processed	
12	Female	50	UL, LL, right	432 g, 21 × 13.5 × 4 cm	Fibrosis, UIP-pattern, antisynthetase Syndrome	20 py until 3 years before surgery	WST-1 and LDH assay, cilia check, histologic evaluation	Tissue stored overnight at 4°C, then processed	
13	Male	59	UL, LL, right	585 g, 22 × 18 × 7 cm	Fibrosis, clinical NSIP	2 py until 37 years before surgery	WST-1 and LDH assay, cilia checks, histologic evaluation	Tissue stored overnight at 4°C, then processed	
14	Male	62	UL, right	556 g, 18.5 × 14 × 3.5 cm	Fibrosis, chronic interstitial pneumonia, UIP pattern	22 py until 22 years before surgery	WST-1 and LDH assays, cilia check, histologic evaluation	Immediate	
15	Male	34	UL, LL, right	582 g, 22.5 × 14.5 × 5 cm	Fibrosis, IPF	No smoking	WST-1 and LDH assays, cilia check, histologic evaluation	Immediate	
16	Female	50	UL, ML, right	389 g, 20 × 12.5 × 3 cm	Fibrosis, UIP pattern, ANCA-positive interstitial pneumonia	No smoking	WST-1 and LDH assays, cilia check, histologic evaluation	Immediate	

ANCA, antineutrophil cytoplasm autoantibodies; IPF, idiopathic pulmonary fibrosis; LDH, lactate dehydrogenase; LL, lower lobe; ML, middle lobe; NSCLC, non–small cell lung cancer; NSIP, nonspecific interstitial pneumonia; py, pack-years; UIP, usual interstitial pneumonia; UL, upper lobe; WST-1, water soluble tetrazolium 1.

### PCLS Generation and Cultivation

Lung lobes were inflated under controlled pressure via the bronchial system with 2% agarose, low-gelling temperature (Sigma-Aldrich, St. Louis, MO) in Dulbecco’s modified Eagle’s medium Nutrient Mixture F-12 Ham (DMEM/F-12 HEPES, no phenol red) from Gibco (Darmstadt, Germany). Agarose-filled lobes were cut into approximately 1-cm-thick slices and macroscopically evaluated by a pulmonary pathologist (C.W. under the supervision of D.D.J.) to exclude neoplasms and infections. Human PCLS (8 mm in diameter and 200 to 300 μm thick) were generated in cold Earle's Balanced Salt Solution (Gibco) using an Alabama RD MD6000 Tissue Slicer (Alabama Research and Development, Munford, AL) as described previously.^12^^,^^13^^,^^38^ Two PCLS per well were incubated in 24-well culture plates (Supplemental Figure S1) with 500 μL of DMEM/F12 supplemented with penicillin (100 U/mL) and streptomycin (100 μg/mL) under standard cell culture conditions (37°C, 5% CO_2_, and 100% humidity), and the medium was changed regularly. Before medium change, each well that contained PCLS was stereoscopically (eightfold to 40-fold magnification) assessed using a Zeiss (Oberkochen, Germany) stereoscope to identify bacterial or fungal contaminations.

### Viability Assays during PCLS Cultivation

Viability of total PCLS was monitored by water soluble tetrazolium (WST)-1 and lactate dehydrogenase (LDH) assays in 96-well plates at days 1, 5, 8, 14, 21, and 28 of cultivation. The cell proliferation reagent WST-1 and Cytotoxicity Detection Kit (LDH) were received from Roche Diagnostics GmbH (Mannheim, Germany). The WST-1 and LDH assays were performed in accordance to the manufacturer's instructions and as described by Neuhaus et al.^38^ As a cell death control, two PCLS per well were previously treated with 1% Triton X-100 for 45 minutes at 37°C, 5% CO_2_, and 100% humidity for each LDH and WST-1 assay. Duplicates of each sample were measured using a Synergy 2 Multi-Mode Microplate Reader (BioTek, Winooski, VT). Most of the PCLS from the assays were also used for the histomorphologic evaluation with respect to tissue integrity.

### Assessment of Cilia Functionality during PCLS Cultivation

The presence or absence of ciliary beat on the airway epithelium of each small airway per PCLS was microscopically assessed every day the medium was changed with a CKX 41 inverse microscope (Olympus, Tokyo, Japan) under 40- and 80-fold magnification and a stereoscope with 8- and 40-fold magnification (Zeiss, Oberkochen, Germany).

### Histomorphologic Analysis

**Figure 1:**
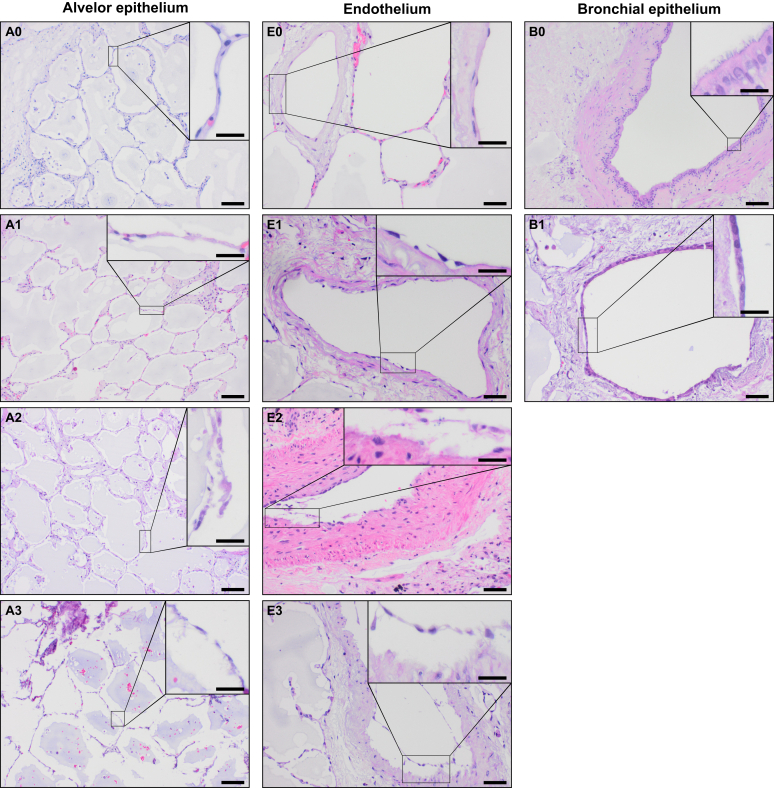
Illustration of the criteria used for histologic grading of human precision-cut lung slices (PCLS). The PCLS and the respective grading are shown broadly and with a magnification (**insets**) of the characteristics concomitant with the grading. A detailed description of the criteria can be found in the text (Materials and Methods). Briefly, the alveolar epithelium was scored from A0 (best) to A3 (worst), the endothelium was scored from E0 (best) to E3 (worst), and the bronchial epithelium was scored from B0 (intact) to B1/2 (detached). Scale bars: 100 μm (**A0–A3** and **B0–B1**); 50 μm (**E0–E3**); 20 μm (**insets**). Original magnifications: ×100 (**A0–A3** and **B0–B1**); ×200 (**E0–E3**); ×400 (**insets**).

After cultivation, specimens were fixed with buffered formalin and subsequently paraffin embedded. Paraffin-embedded specimen were sectioned into 2-μm slices. Sections of PCLS were serially taken from the middle part of the specimen. Subsequently, slices were mounted on object slides and stained with hematoxylin and eosin and with elastic Verhoeff-Van Gieson. Immunohistochemistry was performed according to the diagnostic routine procedure on a Ventana BenchMark ULTRA (Roche, Basel, Swiss). Specimens were stained for p63 (CM 163, diluted 1:100) (Biocare Medical, Pacheco, CA) and TTF1 (M3575, diluted 1:500) (Agilent Dako, Santa Clara, CA) according to the manufacturer's protocols. The histomorphologic pattern with respect to the alveolar and bronchial epithelium as well as the endothelium integrity was evaluated over the entire area of each PCLS by an experienced pulmonary pathologist and a biologist (C.W. and P.B., respectively, under the supervision of D.D.J.) (Figure 1). The alveolar septal structure was scored semiquantitatively as no apparent cell loss within the alveolar septa (score 0), occasional cell loss (score 1), moderate cell loss with many lost alveolar cells (score 2), and severe cell loss when most of the alveolar cells were detached (score 3). The endothelium of arterial and venous vessels were evaluated semiquantitatively as endothelium that is still adherent to the membrana elastica interna or media (score 0), endothelium that is partially (<20% of the endothelium/PCLS is detached) (score 1), moderately (≥20% and ≤50% of the endothelium is detached) (score 2), or strongly affected (>50% of the endothelium is detached) (score 3). The respiratory epithelium of small airways, bronchioles, and bronchi was graded as B0 if it was completely adherent at the lamina propria or tunica adventitia, as B1 if detached once in consecutive sections, and as B2 if detached in more than one airway per PCLS.

### Transcriptome Analysis Using RNASeq

RNA was isolated using the Maxwell RSC simplyRNA Cells Kit (Promega, Madison, WI) with the Maxwell RSC Instrument as described in the instruction manual. PCLS were homogenized in 200 μL of chilled 1-thioglycerol/homogenization solution by sonification under cold conditions. Quality and integrity of total RNA were controlled on Agilent Technologies 2100 Bioanalyzer (Agilent Technologies, Santa Clara, CA). The mean RNA quality number was 9.5, with the lowest number being 7.4. The RNASeq library was generated from 100 ng of total RNA using NEBNext Single Cell/Low Input RNA Library Prep (New England BioLabs, Ipswich, MA) according to the manufacturer's protocols. The libraries were sequenced on Illumina NovaSeq 6000 (Illumina, San Diego, CA) using the NovaSeq 6000 S2 Reagent Kit (100 cycles, paired end run) with a mean of 3 × 10^7^ reads per RNA sample. Each FASTQ file gets a quality report generated by FASTQC tool (FastQC, Babraham Institute, S. Andrews, *https://www.bioinformatics.babraham.ac.uk/projects/fastqc*, last accessed February 10, 2021). Before alignment to the reference genome, each sequence in the raw FASTQ files was trimmed on base call quality and sequencing adapter contamination using the Trim Galore! wrapper tool (Trim Galore!, Babraham Institute, F. Krueger, *https://www.bioinformatics.babraham.ac.uk/projects/trim_galore*, last accessed February 10, 2021). Reads shorter than 20 bp were removed from the FASTQ file. Trimmed reads were aligned to the reference genome using the open source short read aligner STAR (*https://code.google.com/p/rna-star*, last accessed February 10, 2021) with settings according to the log file.^39^ Feature counts were determined using the R package Rsubread.^40^ Only genes showing counts >5 at least two times across all samples were considered for further analysis (data cleansing). Gene annotation was performed with the R package bioMaRt.^41^ Before starting the statistical analysis steps, expression data were log_2_ transformed and TMM normalized (edgeR).^42^ Differential gene expression was calculated by the R package edgeR. Functional analysis was performed by the R package clusterProfiler.^43^

### Statistical Analysis

Reported means and SDs were weighted (ie, adjusted) according to the number of PCLS analyzed from each tissue source and point of time so that each measurement contributes equally regardless of the number of PCLS analyzed. For statistics and modeling purposes of viability measurements, linear mixed-effect models were used and fitted via maximum likelihood and weighted according to the number of PCLS using the R packages lme4 version 1.1-23 and lmerTest version 3.1-2. The parameters tissue type, tissue processing, and time of cultivation were used as fixed effects, whereas individual lung identifiers were designated as random effects to account for any bias introduced by the individual specimen. All reported *P* values for parameters were calculated from the *t* statistics from this type of models, with the selection of samples and parameters depending on the question at hand.

## Results

More than 1500 human PCLS from 16 different donors (seven fibrotic lung explants and nine lung tumor resections) were cultivated for up to 28 days to determine the duration for which the tissue structure and functionality as well as the cell viability were preserved (Table 1). Furthermore, changes in transcriptome during cultivation of human PCLS derived from lung tumor resections (*n* = 3) were analyzed. PCLS derived from lung tumor resection material were generated from tumor-free tissue. To investigate the effect of overnight storage of lung tissue at 4°C on the viability and integrity of PCLS, specimens of six donors (two resections of lung tumors and four fibrotic lung explants) were maintained for up to 17 hours at 4°C (means ± SD time of 4°C storage: 14 ± 4 hours) either filled with low-gelling agarose or unfilled (Table 1), before further processing. Because of low sample size of overnight stored lung tumor resection tissue (*n* = 2) and the fact that PCLS from overnight stored lung tumor resections did not significantly differ throughout all analyses, all lung tumor resections were considered as a homogenous group.

### Viability and Cytolysis of Long-Term Cultivated Human PCLS

**Figure 2:**
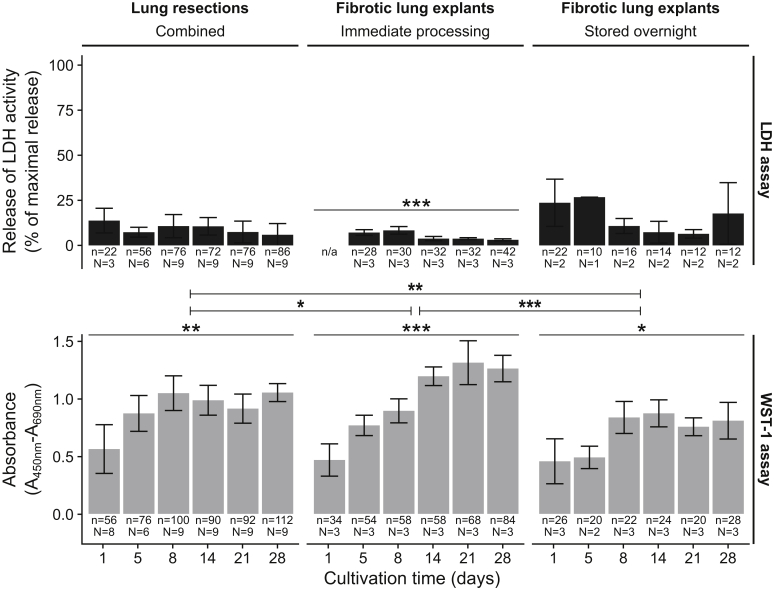
Viability of long-term cultivated human precision-cut lung slices (PCLS) as measured by the water soluble tetrazolium (WST)-1 (for quantification of cell proliferation and viability) and lactate dehydrogenase (LDH) (for quantification of cell death and cell lysis) assays. PCLS were derived from immediately processed or overnight stored fibrotic explants as well as tumor lung resections. WST-1 values are shown as the corrected absorbance, and LDH values are represented as the relative amount of released LDH activity compared with the totally lysed control. Data are expressed as means ± SD. ∗*P* < 0.05, ∗∗*P* < 0.01, and ∗∗∗*P* < 0.001. n, number of PCLS; N, number of lungs.

Viability of 1, 5, 8, 14, 21, and 28 days cultivated PCLS (*n* = 1660) was determined by the colorimetric cell proliferation reagent WST-1–based assay, which is commonly used for spectrophotometric quantification of cell proliferation, growth, and viability of cultured cells, including PCLS.^12^^,^^13^^,^^38^ In addition, cell death and cell lysis during long-term cultivation was measured by LDH activity because LDH is released into the supernatant by damaged cells. The LDH assay is a widely used standard method to quantify the extent of cell lysis in relation to a positive control (100% cells are lysed) of human PCLS.^12^^,^^13^^,^^38^ PCLS cultivated in serum-free conditions were viable for up to 28 days (Figure 2).

Overall, PCLS from immediately processed fibrotic explants and lung tumor resections showed LDH assay values <20% of the detergent (1% Triton X-100) lysed positive control (weighted means ± SD, 7.77% ± 5.65%; median, 5.7%; *n* = 552 PCLS) and absorbance values in the WST-1 assays (weighted means ± SD, 0.9 ± 0.26; median, 0.94; *n* = 882 PCLS) (Figure 2) that are in accordance with other studies regarding long-term cultivation of human PCLS during a 14-day period.^12^ The lowest WST-1 values were observed after 1 day of cultivation, but increasing values during cultivation indicated a recovery of viability after the initial procedure of PCLS generation.^12^^,^^13^ Viability values in immediately processed fibrotic lung tissue (WST-1 weighted means ± SD, 1.03 ± 0.3; *n* = 356 PCLS) were slightly but significantly (*P* = 4.06 × 10^−02^) better when compared with lung resection tissue (WST-1 weighted means ± SD, 0.92 ± 0.2; *n* = 526 PCLS) (Figure 2).

PCLS from all tissue types showed a significant (*P* ≤ 2.25 × 10^−02^) increase in viability as measured by WST-1 assays during long-term cultivation over 28 days (Figure 2). An overnight storage of fibrotic lungs before agarose filling led to a significant (*P* = 1.55 × 10^−04^) loss in viability (WST-1 weighted means ± SD, 0.69 ± 0.21; *n* = 140 PCLS) compared with the immediately processed samples (WST-1 weighted means ± SD, 1.03 ± 0.30; *n* = 356 PCLS).

### Ciliary Beat in the Small Airways of Long-Term Cultivated Human PCLS

**Figure 3:**
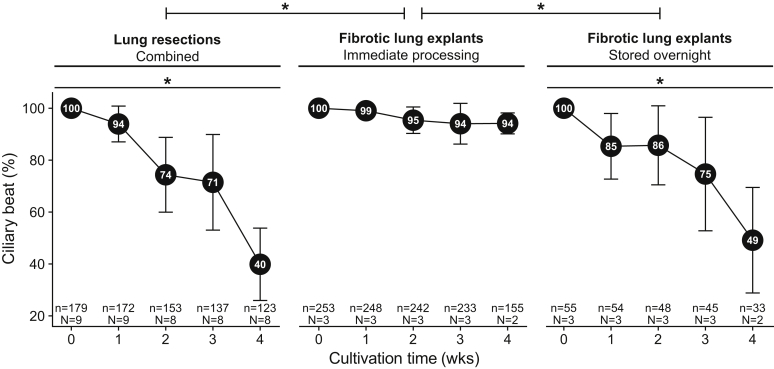
Preservation of ciliary beat in human precision-cut lung slices (PCLS) during long-term cultivation. The PCLS derived from immediately processed or overnight stored fibrotic explants as well as tumor lung resections. Ciliary beat was assessed on each day of medium change, and the relative number of PCLS with ciliary beat was calculated compared with the initial number of PCLS with ciliary beat. Weighted means ± SD were used for the statistical analysis of all data points. Graphs were generated from weighted means of a limited number of data points (week 1 = day 7 ± 1, week 2 = day 14 ± 1, week 3 = day 21 ± 1, week 4 = day 28 ± 1) for illustration purposes. Data are expressed as means ± SD. ∗*P* < 0.05. n, number of PCLS; N, number of lungs.

The ciliary beat of PCLS (*n* = 487) originating from 15 different donor lung tissues (six fibrotic explants and nine lung tumor resections) cultured for 28 days was regularly recorded before every medium change. Eighteen PCLS (means ± SD, 18.4 ± 6.9) from lung tumor resections, 78 PCLS (means ± SD, 78.1 ± 21.0) from immediately processed fibrotic explants, and 17 (means ± SD, 17.4 ± 10.6) from overnight stored fibrotic explants could be used for observation of ciliary beat on each recording day. During 4 weeks of cultivation, a gradual loss (*P* = 2 × 10^−16^, *n* = 15 lungs) of the ciliary beat in the small airways of human PCLS was observed (Figure 3). Overnight storage of fibrotic lungs led to a significantly higher loss of ciliary beat over time when compared with immediately processed fibrotic explants (*P* = 3.68 × 10 ^−02^). After 1 week of culture, most of the initially observed ciliary beat in PCLS could still be observed regardless of the sample type (lung resection specimens weighted means ± SD, 93.9% ± 6.9%; immediately processed fibrotic specimens weighted means ± SD, 99.0% ± 0.6%; overnight stored fibrotic specimens weighted means ± SD, 85.3% ± 12.6%). The amount of PCLS from lung resections with ciliary beat was reduced to 71.5% ± 18.4% after 3 weeks of culture, and after 4 weeks only 39.9% ± 13.9% of the initially observed ciliary beat could be observed. Interestingly, in PCLS from immediately processed fibrotic tissue, 94.2% ± 4.0% of the ciliary beat could still be observed after 4 weeks of culture. In stark contrast, PCLS that originated from overnight stored fibrotic specimen showed a strong reduction of ciliary beat after 4 weeks of culture (weighted means ± SD, 49.1% ± 20.3%), indicating a strong effect of overnight storage on ciliary beat in fibrotic PCLS. These data indicate that >90% of ciliary beat is preserved for at least 1 week of cultivation of immediately processed lung tissue.

### Histomorphologic and Pathologic Changes in Long-Term Cultivated Human PCLS

A total of 1518 human PCLS from 16 donors (seven fibrotic lung explants and nine lung tumor resections) were hematoxylin and eosin and elastic Verhoeff-Van Gieson stained, and the integrity of the alveolar, blood vessel, and airways structure was microscopically assessed at days 1 (*n* = 189), 5 (*n* = 217), 8 (*n* = 257), 14 (*n* = 249), 21 (*n* = 266), and 28 (*n* = 340) of cultivation, respectively. The bulk of these PCLS was previously analyzed with respect to viability and cytolysis by WST-1 and LDH assays.

**Figure 4:**
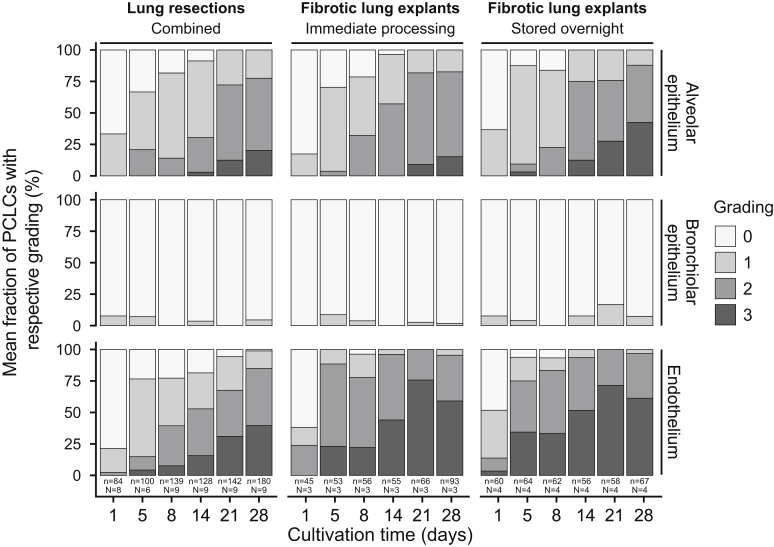
Histomorphologic changes in long-term cultivated human precision-cut lung slices (PCLS) derived from immediately processed or overnight stored fibrotic explants as well as tumor lung resections. Morphologic changes were analyzed considering the three major structural compartments of a lung slice (alveolar epithelium, bronchial epithelium, and endothelium). The grading ranges from 0 to 3 (bronchial epithelium, 0 to 2), with 0 displaying the best and 3 the worst condition (a detailed description can be found in Materials and Methods). Each day shows the weighted mean fraction of PCLS with respective grading. n, number of PCLS, N, number of lungs.

Integrity of the alveolar and endothelium structure was maintained after 1 day of cultivation (Figure 4). As of day 5, a slight loss of pneumocytes and a slight to moderate detachment of the endothelium in blood vessels emerged in most PCLS.

Although most PCLS derived from lung tumor resections showed a moderately detached endothelium (grade 2) from day 14 to 28 of cultivation, most fibrotic PCLS showed a severely detached endothelium (grade 3). In addition, in most fibrotic PCLS, a moderate loss of pneumocytes in the alveolar epithelium (grade 2) was present from day 14 onward (Figure 4). In PCLS derived from lung tumor resections, the moderate loss of pneumocytes (grade 2) abundantly occurred from day 21 onward. Overall, a significant loss of the alveolar epithelium integrity (*P* < 2 × 10^−16^) and a significant detachment of the endothelium (*P* = 3.95 × 10^−14^) could be observed during 4 weeks of cultivation. PCLS from immediately processed or overnight stored fibrotic explants were significantly more affected regarding the detachment of the endothelium (*P* = 1.37 × 10^−07^) compared with PCLS derived from lung tumor resections during long-term cultivation of PCLS. PCLS from freshly processed or overnight stored fibrotic explants did not differ significantly with respect to alveolar (*P* = 0.31) and endothelium (*P* = 0.68) degeneration. The integrity of the bronchiolar epithelium remained well preserved for up to 28 days in culture regardless of the tissue type or processing (Figure 4).

**Figure 5:**
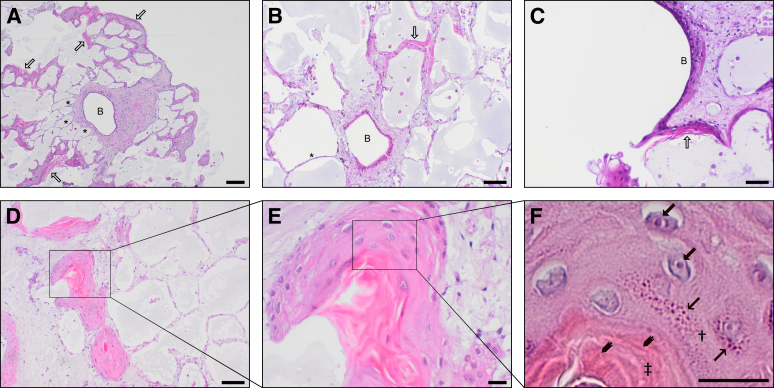
Exemplary illustration of long-term cultivated human precision-cut lung slices (PCLS) with foci of squamous metaplasia predominantly found in the alveolar epithelium and rarely adjacent to bronchial epithelium. **A:** Low-power magnification of a PCLS severely affected by keratinizing squamous metaplasia (**hollow arrows**) after 28 days of cultivation. Metaplasia is found in the alveolar epithelium distal from normal-appearing bronchiole (B) and with normal-appearing alveolar septum (**asterisks**) in between. **B:** Image section of a normal-appearing bronchiole (B) next to a normal-appearing alveolar septum (**asterisks**) and distal to alveolar epithelium affected by squamous metaplasia (**hollow arrow**). **C:** High-power magnification of a bronchiole (B) with absent cilia and a transition to keratinizing squamous metaplasia (**hollow arrow**). **D:** Overview of a PCLS with apparent foci of squamous metaplasia and keratinization. **E:** High-power magnification of squamous metaplasia with prominent keratinization. **F:** Higher magnification with narrowed condenser aperture showing the characteristic features of squamous metaplasia with keratinization: Prominent nucleoli (**arrows**), keratohyaline granules (**spiky arrows**), corneocytes (**directional arrows**), stratum granulosum (**single dagger**), and stratum corneum (**double dagger**). Scale bars: 200 μm (**A**); 100 μm (**B** and **D**); 50 μm (**C**); 20 μm (**E** and **F**). Original magnifications: ×40 (**A**); ×100 (**B** and **D**); ×200 (**C**); ×400 (**E** and **F**).

**Figure 6:**
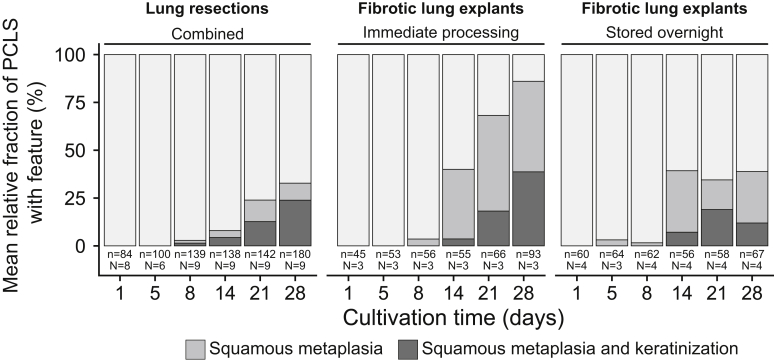
Occurrence of squamous metaplasia and keratinization in long-term cultivated human precision-cut lung slices (PCLS) derived from immediately processed or overnight stored fibrotic explants as well as tumor lung resections. Each day shows the weighted mean fraction of PCLS in which squamous metaplasia and keratinization occurred. Keratinization was never observed without the occurrence of squamous cell metaplasia. n, number of PCLS; N, number of lungs.

Interestingly, squamous metaplasia of the alveolar epithelium and subsequent keratinization (Figure 5) were observed. Supplemental Figure S2 shows the foci of alveolar squamous metaplasia found in long-term cultivated PCLS compared with alveolar squamous metaplasia found in a lung tissue sample from an autopsy. Metaplastic foci of both specimens were checked for malignant neoplasm as evaluated by the expression pattern of p63 and TTF1. In both cases, the presence of basal epithelial cells as measured by immunohistochemistry (p63) was demonstrated, opposing malignant neoplasia. Furthermore, a loss of TTF1 in squamous metaplasia cells in both specimens in line with epithelial metaplasia toward a squamous cell–like phenotype was shown. Squamous metaplasia in both specimens were nonneoplastic as evaluated by an experienced pathologist (C.W. under the supervision of D.D.J.). Squamous metaplasia was found in PCLS derived from all donor specimens, with varying degree and starting times (Figure 6). Foci of squamous metaplasia were present in 328 of 1518 PCLS (22%), and in 92% of the cases, they originated from the periphery of the PCLS. These foci were first observed at day 5 of cultivation, at which time the number of affected PCLS increased significantly (*P* < 2 × 10^−16^) over time (Figure 6). Squamous metaplasia was more than twice as frequent in PCLS derived from immediately processed fibrotic lung explants [149 of 368 PCLS (41%)] as in PCLS derived from overnight stored fibrotic lung explants [71 of 367 PCLS (19%), *P* = 6.74 × 10^−02^] or lung tumor resections [108 of 783 PCLS (14%), *P* = 3.33 × 10^−08^]. At the end of cultivation (day 28), 86% of the PCLS from immediately processed fibrotic lungs, 39% of PCLS from overnight stored fibrotic lungs, and 33% of PCLS from lung tumor resections were affected by squamous metaplasia.

A keratinization of the squamous epithelium was detected in 142 of 1518 PCLS (9.35%) with a significantly increasing prevalence during cultivation (*P* = 7.51 × 10^−15^) (Figure 6). Initial keratinization of the alveolar or bronchial epithelium was first observed at day 8 of cultivation in PCLS derived from lung tumor resections and as of day 14 in PCLS derived from fibrotic lung explants (Figure 6). Although keratinization occurred in 50 of 368 PCLS (13.59%) derived from immediately processed fibrotic lungs, it was only visible in 23 of 367 PCLS (6.27%) derived from overnight stored fibrotic lungs. In PCLS that originated from lung tumor resections, keratinization was identified in 69 of 783(8.81%).

### Changes in the Transcriptome of PCLS during Long-Term Cultivation

**Figure 7:**
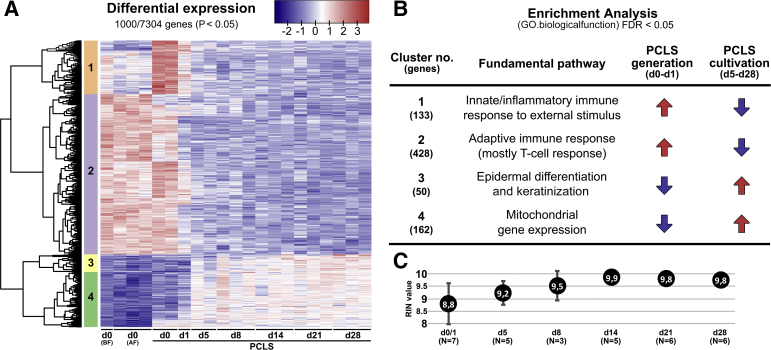
Transcriptome analysis of long-term cultivated human precision-cut lung slices (PCLS) using RNA sequencing and pathway enrichment analysis. The PCLS used for sequencing originated from three tumor resection lungs. **A:** Heat map and clustering of genes calculated from the differential expression of the top 1000 genes. Every lane represents a different lung specimen and/or a different day of sampling. In addition, samples were taken from lungs before filling with agarose (d0 BF) and after filling with agarose (d0 AF). **B:** Enrichment analysis [Gene Ontology (GO): biological function] of the gene sets belonging to the respective cluster for the identification of overrepresented pathways (Supplemental Figure S3). The number of genes significantly associated with the respective pathways of a cluster are written in brackets. On the basis of the identified pathways, each cluster was manually assigned to a higher-order category. **C:** RNA integrity number (RIN) of RNA isolated from PCLS (fibrotic and tumor lung resections) at different points in time during long-term cultivation. FDR, false discovery rate; N, number of lungs.

To investigate changes in the transcriptome of PCLS during long-term cultivation, RNASeq analysis on up to three different lung tumor resections samples per time point was performed. In addition, samples taken before and after agarose filling of the lung specimen were analyzed to investigate possible effects of the filling procedure onto the transcriptome. Gene mapping identified a total of 7304 differentially regulated genes. A heatmap from the top 1000 differentially expressed genes is reported by edgeR (Figure 7A). Four major clusters of gene sets could be identified. For each cluster the significantly overrepresented pathways were determined by pathway enrichment analysis (Figure 7B; Supplemental Figure S3). The enriched pathways in every cluster were manually assigned to a higher-order category of biological function. Significantly overrepresented pathways of the innate, inflammatory, and adaptive immune response were found and were generally up-regulated during and shortly after the PCLS generation procedure (Figure 7, cluster 1 and 2). In particular, the pathways associated with the T-cell response were up-regulated before and after lung filling as well as in PCLS at the day of generation (Figure 7, cluster 2). Interestingly, expression of genes associated with pathways of the innate and inflammatory immune response peaked shortly after PCLS generation on day 0 of cultivation (Figure 7, cluster 1). Gene set expression levels associated with innate and adaptive immune responses had already decreased on day 5 and stayed at a low level during additional cultivation. Another cluster included overrepresented pathways associated with mitochondrial gene expression (Figure 7, cluster 4). Most genes in these pathways were significantly up-regulated in PCLS between 5 and 28 days of cultivation compared with samples harvested before and after lung filling as well as in PCLS during the first 24 hours of cultivation. Gene sets of the overrepresented pathways in cluster 3 showed a similar pattern as cluster 4 during cultivation (Figure 7, cluster 3). Overrepresented pathways of cluster 3 include pathways that can be manually assigned to processes associated with epidermal differentiation and keratinization. Genes included in these pathways were mostly up-regulated during cultivation when compared with day 0 (Figure 7, cluster 3).

## Discussion

Human PCLS as *ex vivo* three-dimensional cell culture models are widely used to study the pathophysiology, mechanisms, and therapy of different lung diseases because PCLS highly resemble the original anatomical and biological tissue environment with respect to cell-cell and cell-matrix interactions.^5^^,^^7^^,^^8^^,^^44^ A challenge associated with PCLS-based analyses is to maintain its integrity *ex vivo*. Most toxicologic and pharmacologic studies do not depend on prolonged cultivation,^7^^,^^17^^,^^34^^,^^35^ but future approaches of modeling complex disease mechanisms or analyzing therapeutic modes of action might need cultivation times beyond 1 week. PCLS cultures submerged in antibiotic-supplemented DMEM are viable for up to 15 days.^5^^,^^12^^,^^36^^,^^38^ However, some changes in histology and functionality appear to manifest.^12^^,^^38^ During long-term cultivation for up to 15 days, a general reduction in cytokine and chemokine production, increased inflammatory activity, a slight separation of connective tissue fibers, a decreased number of pneumocytes, and a reduction in methacholine-induced bronchoconstriction have been described so far.^5^^,^^12^^,^^13^^,^^36^

**Figure 8:**
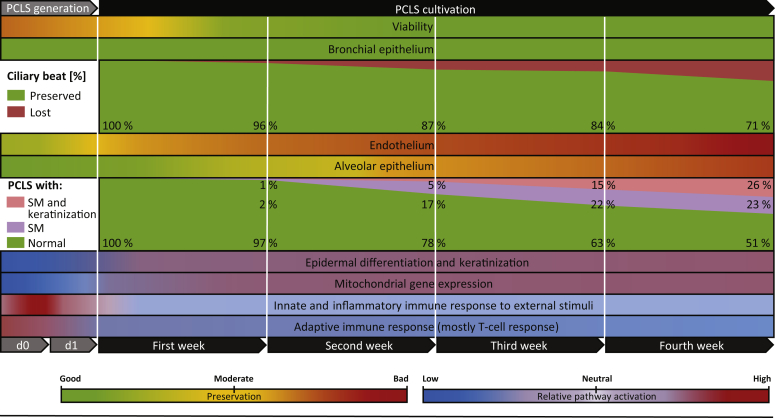
Abstraction of all findings, including all data regardless of the tissue type (fibrotic or tumor resection lungs) or processing (immediate processing or overnight storage). Viability (measured by lactate dehydrogenase and water soluble tetrazolium-1 assays), tissue integrity (endothelium and bronchial and alveolar epithelium), and transcriptome analysis (RNA sequencing pathway enrichment analysis) was visualized manually. The graphs for ciliary beat and the histopathologic findings [squamous metaplasia and keratinization] were generated from weighted means, including precision-cut lung slices (PCLS) from all conditions (ciliary beat: N/day = 13 to 15, n/day = 386 to 487; histopathologic findings: N/day = 16, n/day = 249 to 340) at the specific point in time (ciliary beat: days 0, 7 ± 1, 14 ± 1, 21 ± 1, and 28 ± 1; histopathologic findings: days 1, 8, 14, 21, and 28). n, number of PCLS; N, number of lungs.

In this study, the cultivation time of human PCLS was extended to 28 days, and ongoing histologic, transcriptomic, and functional changes were quantified to an unprecedented extent to comprehensively evaluate the preservation of different compartments in tissue slices and address histopathologic changes occurring during long-term cultivation under serum-free conditions. Although several studies describe well-preserved tissue integrity and viability of long-term cultivated lung tissue slices,^12^^,^^13^^,^^36^^,^^45^^,^^46^ histomorphologic and histopathologic changes appearing with different grades of severity and onset points in the anatomical compartments of PCLS over time could be elucidated. Although overall viability is well preserved during long-term cultivation (4 weeks), the number of PCLS with ciliary beat is gradually decreasing after 1 week. Tissue structure integrity suffers from continuous cell loss over time and is accompanied by an increasing manifestation of squamous metaplasia and keratinization (Figure 8).

One day after PCLS generation, all specimens exhibited reduced metabolic activity (as measured by WST-1 assay) attributable to ischemia and shear stress during processing. Reduced metabolic activity is also indicated by a significant decrease in mitochondrial activity (as measured by RNASeq) during the first 24 hours of cultivation. After 1 week, all PCLS, irrespective of tissue type (fibrotic versus lung tumor resection specimens) or processing (stored overnight versus immediate processing), recovered and showed viability values comparable to those reported in the literature for this particular set-up.^13^ PCLS stayed viable for the whole time of cultivation.

Histomorphologic integrity was highly dependent on tissue type, processing time, and the respective anatomical compartment. In general, alveolar and vasculatory integrity significantly decreased over time, and PCLS generated from fibrotic lung tissue were more severely affected than PCLS generated from lung tumor resections. Although the alveolar epithelium of PCLS from lung tumor resections remained mostly intact until day 21, most PCLS from fibrotic lung tissue exhibited a moderate loss of pneumocytes as early as day 14. The loss of integrity was strongest in the blood vessels, where most of the fibrotic PCLS showed a moderate to severe detachment of the endothelium already within the first week of cultivation. In contrast, most of the PCLS from lung tumor resections only showed a slightly detached endothelium until day 14. The observation that the integrity of the vascular compartment is lost first in PCLS has also been made by Siminski and colleagues,^45^ and it has been proposed that this might be prevented by inflating the vasculatory system with supporting agarose. Indeed, the vasculatory system is physiologically under constant pressure via the blood in the circulatory system. Taking away the pressing stimulus likely contributes to the collapse of the endothelial structure and might therefore be prevented by structure-supporting agarose. Of all compartments, the bronchial tree showed the best preservation on a morphologic level. During cultivation, the bronchial epithelium exhibited almost no apparent loss in structure or cell integrity, irrespective of tissue type or processing. Nevertheless, cilia functionality was checked during cultivation. On the basis of these findings, it was concluded that studies regarding the ciliary beat can be conducted for at least 1 week because a ciliary beat is still visible in >90% of the PCLS from immediately processed tissue. The ciliary beat in a given PCLS significantly decreases beyond 1 week of cultivation. Interestingly, after 4 weeks, >90% of PCLS from immediately processed fibrotic lungs, and <50% of PCLS from lung tumor resections or overnight stored fibrotic tissue, exhibited a ciliary beat. Although no plausible explanation for this phenomenon could be found, high heterology between samples from different patients must always be taken into account.

A rather unexpected finding was the occurrence of squamous metaplasia and keratinization, a pathologic transformation that, to our knowledge, has previously not been described in the context of human PCLS cultivation. However, it was observed as a secondary finding in the context of fibrotic remodeling or diffuse alveolar damage in human lung specimen (Supplemental Figure S2). In most cases, metaplasia was found in the alveolar epithelium (Figure 5A and B), where a transformation of pneumocytes into keratinocytes, identified by characteristic features such as a prominent nucleolus, keratohyaline granules, and terminal differentiation into corneocytes, was observed (Figure 5F). Squamous metaplasia was found in PCLS of every patient, regardless of the clinical background, and first occurred after 1 week of cultivation, while significantly increasing over time. Metaplasia is a transformation of one differentiated cell type to another related type not normally present in a specific tissue.^47^^,^^48^ It is the result of an altered gene expression in response to, for example, chronic physiologic, chemical, or physical irritation.^48^, ^49^, ^50^, ^51^ The resulting phenotype is more resistant but loses its original function.^47^^,^^48^ Immunohistochemical analysis of apparent foci of squamous metaplasia in long-term cultivated PCLS showed no features of malignant neoplasm. Therefore, keratinizing squamous metaplasia is most likely a somewhat delayed response to extensive injury caused by the cutting procedure because in 92% of cases metaplasia originated from the periphery of the PCLS. This finding provides the possibility of using PCLS as an *ex vivo* model to study mechanisms of adaption to acute lung injury. Another possible reason for the metaplastic changes might be the lack of vitamin A in the cultivation medium, even though a correlation between vitamin A deficiency and squamous metaplasia has only been described in organ culture of hamster trachea.^49^ Nevertheless, structural analogs of vitamin A are able to reverse metaplastic changes, which might also be beneficial for metaplastic changes observed in PCLS culture.^49^^,^^52^ Metaplasia of the alveolar epithelium most likely originates from type 2 pneumocytes because those serve as progenitor cells of type 1 pneumocytes during physiologic parenchymal homeostasis and after injury.^53^, ^54^, ^55^ In addition, Wang and colleagues^56^ found that 95% of type 1 pneumocytes in mice are terminally differentiated (not able to transdifferentiate and proliferate anymore). However, type 1 cells are also able to transdifferentiate into type 2 cells.^57^ PCLS could well serve as a model to examine pulmonary repair mechanisms and elucidate the origin and stages of cell differentiation during these processes. After 4 weeks of cultivation, 86% of immediately processed fibrotic PCLS were affected by squamous metaplasia, which occurred twice as frequently as in overnight stored fibrotic tissue and tumor lung resections. Fibrotic PCLS are likely more severely affected because of an abnormal mesenchyme with hyperactive myofibroblasts and dysregulated repair mechanisms found in pulmonary fibrosis.^58^ Long-term cultivated fibrotic PCLS may therefore be of considerable use as a model to elucidate dysregulated repair mechanisms.

In summary, PCLS experiments should not be started before a resting phase of at least 24 hours because of significantly reduced metabolic activity and a significantly increased inflammatory response during that period. Khan et al^36^ even suggest a resting phase of at least 4 days because of inflammatory activity. Furthermore, the bronchial epithelium suffered from no apparent cell loss, but cilia functionality markedly decreased after the first week of cultivation. Most importantly, exclusively checking viability values of human PCLS can be delusive. Even though viability significantly increased over time, it was accompanied by continuous loss of pneumocytes and endothelial cells. Although the alveolar epithelium was mostly preserved for 2 weeks, the vascular system suffered from severe cell loss already within the first week. At first glance, there seems to be a discrepancy between increasing viability and cell loss. However, the lost pneumocytes and endothelial cells account for only a small portion of all cells present in a lung slice. Most cells can be found in the bronchial epithelium and interstitium. Furthermore, the increasing number of metaplastic cells might compensate or even overcompensate for the loss of cells, thereby artificially increasing viability values of PCLS. Because PCLS from immediately processed fibrotic lung tissue showed twice as many metaplastic changes as PCLS from lung tumor resections, it would also explain why fibrotic PCLS had significantly higher viability values while at the same time suffering from more severe cell loss than PCLS from lung tumor resections. In addition, it has to be considered that squamous metaplasia is also a known preneoplastic change in the bronchial epithelium.^59^^,^^60^ Metaplastic changes that were observed here were mostly present in the alveolar epithelium. Nevertheless, in future approaches of modeling lung cancer development in *ex vivo* tissue cultures, one needs to cautiously distinguish between preneoplastic changes and squamous metaplasia that is caused by cutting damage and developing during cultivation. Finally, it is unclear whether the cell loss, decreasing cilia functionality, and increasing manifestation of metaplasia remain the only functional changes beyond 14 days of cultivation and will be investigated in further studies.

## Supplemental Data

Supplemental Figure S1 **A:** Precision-cut lung slices (PCLS) in a 24-well with medium during cultivation (top view). **B:** Low-power stereoscopic image of a PCLS in a 24-well showing the three-dimensional structure of the lung parenchyma. Original magnification, ×40 (**B**).

Supplemental Figure S2 **A**–**C:** Lung tissue from a patient with fibrotic remodeling and diffuse alveolar damage (autopsy specimen). **D**–**F:** Alveolar squamous metaplasia (SM) found *ex vivo* in precision-cut lung slices (PCLS). Both specimens were stained with hematoxylin and eosin (H&E) as well as immunohistochemically stained for the expression of p63 and TTF1 (data not shown). Positive cells appear dark brown as a result of the horseradish peroxidase assay. p63 was used as a marker for the basal cell layer (**arrows**). **B** and **E**: Both PCLS and lung tissue from an autopsy showed apparent foci of squamous metaplasia. **C** and **F**: In both specimens, the basal cell layer (**arrows**) was positive for p63. TTF1 was negative in basal as well as in luminal SM cells of both specimens (data not shown). Staining pattern of H&E, p63, and TTF1 furnish evidence of apparent foci of squamous metaplasia without signs of neoplasia. Scale bars = 50 μm (**A** and **D**); 25 μm (**B**, **C**, **E**, and **F**). Original magnification: ×50 (**A** and **D**); ×200 (**B**, **C**, **E**, and **F**).

Supplemental Figure S3 Transcriptome analysis of long-term cultivated human precision-cut lung slice (PCLS) using RNA sequencing and pathway enrichment analysis. The PCLS used for sequencing originated from three tumor resection lungs. Overrepresented pathways were identified via enrichment analysis of the gene sets belonging to the respective cluster [Gene Ontology (GO): biological function]. FDR, false discovery rate.
