# Supplementary figures and images for: The Challenge of Long-Term Cultivation of Human Precision-Cut Lung Slices
Source: Am J Pathol. 2022 Feb;192(2):239–53. doi: 10.1016/j.ajpath.2021.10.020 (PMC8891143; doi:10.1016/j.ajpath.2021.10.020)

**A**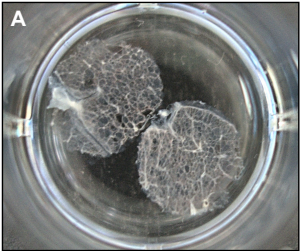**B**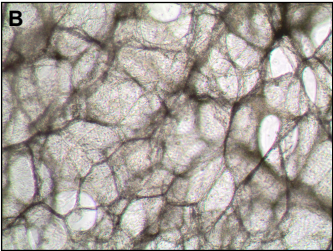

Supplement: Supplemental Figure S1 — A: Precision-cut lung slices (PCLS) in a 24-well with medium during cultivation (top view). B: Low-power stereoscopic image of a PCLS in a 24-well showing the three-dimensional structure of the lung parenchyma. Original magnification, ×40 (B). [file mmc1.pdf]

Lung tissue  
from autopsy

H&E

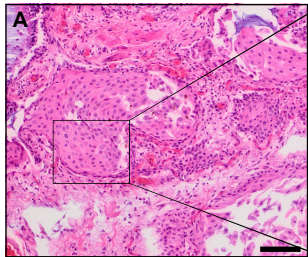

H&E

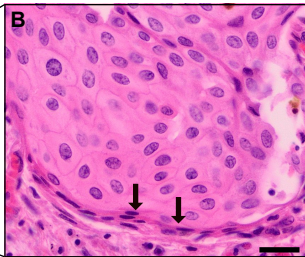

p63

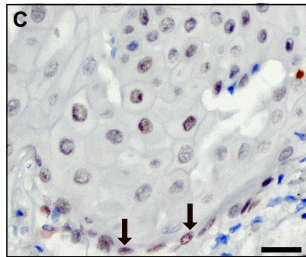

PCLS

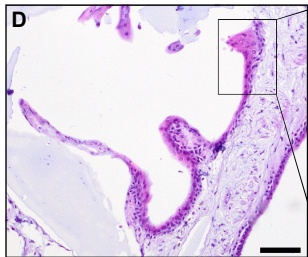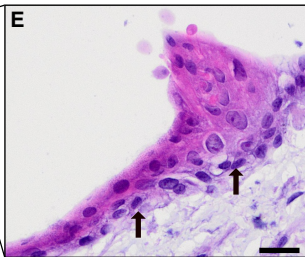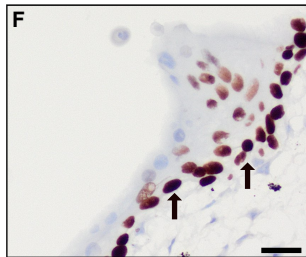

Supplement: Supplemental Figure S2 — A–C: Lung tissue from a patient with fibrotic remodeling and diffuse alveolar damage (autopsy specimen). D–F: Alveolar squamous metaplasia (SM) found ex vivo in precision-cut lung slices (PCLS). Both specimens were stained with hematoxylin and eosin (H&E) as well as immunohistochemically stained for the expression of p63 and TTF1 (data not shown). Positive cells appear dark brown as a result of the horseradish peroxidase assay. p63 was used as a marker for the basal cell layer (arrows). B and E: Both PCLS and lung tissue from an autopsy showed apparent foci of squamous metaplasia. C and F: In both specimens, the basal cell layer (arrows) was positive for p63. TTF1 was negative in basal as well as in luminal SM cells of both specimens (data not shown). Staining pattern of H&E, p63, and TTF1 furnish evidence of apparent foci of squamous metaplasia without signs of neoplasia. Scale bars = 50 μm (A and D); 25 μm (B, C, E, and F). Original magnification: ×50 (A and D); ×200 (B, C, E, and F). [file mmc2.pdf]

Enrichment Analysis (GO.BiologicalFunction) FDR < 0.05

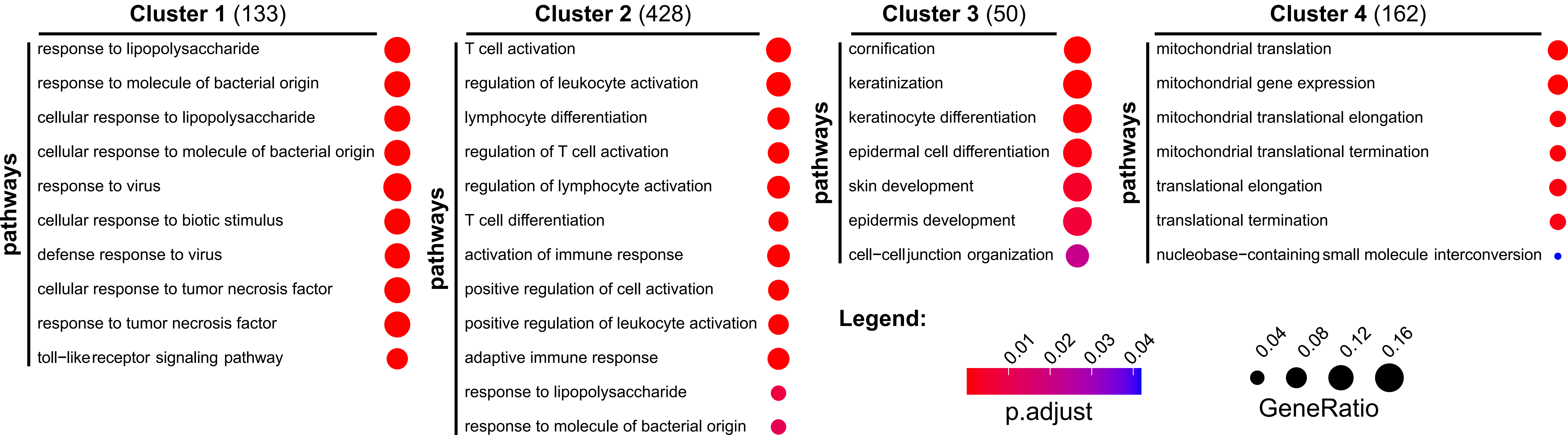

Supplement: Supplemental Figure S3 — Transcriptome analysis of long-term cultivated human precision-cut lung slice (PCLS) using RNA sequencing and pathway enrichment analysis. The PCLS used for sequencing originated from three tumor resection lungs. Overrepresented pathways were identified via enrichment analysis of the gene sets belonging to the respective cluster [Gene Ontology (GO): biological function]. FDR, false discovery rate. [file mmc3.pdf]
